# Causality and preventability assessment of adverse drug events of antibiotics among inpatients having different lengths of hospital stay: a multicenter, cross-sectional study in Lahore, Pakistan

**DOI:** 10.1186/s40360-018-0222-5

**Published:** 2018-06-25

**Authors:** Anum Saqib, Muhammad Rehan Sarwar, Muhammad Sarfraz, Sadia Iftikhar

**Affiliations:** 1 Department of Pharmacy, The Islamia University of Bahawalpur, Bahawalpur, Punjab Pakistan; 2 Akhtar Saeed College of Pharmaceutical Sciences, Lahore, Pakistan; 3 College of Pharmacy, Al Ain University of Science and Technology, Al Ain, Abu Dhabi UAE

**Keywords:** Antibiotics, Adverse drug reactions, Adverse drug events, Length of stay, Causality, Preventability

## Abstract

**Background:**

A large number of hospital admissions are attributed to adverse drug reactions (ADRs) and they are the fifth leading cause of death worldwide. The present study aimed to assess the causality and preventability of adverse drug events (ADEs) of antibiotics among inpatients having different lengths of hospital stay.

**Methods:**

A prospective, observational study was conducted in four tertiary-care public sector hospitals of Lahore, Pakistan. Study population consisted of hospitalized patients who were prescribed one or more antibiotics. Data were collected between 1st January, 2017 and 30th June, 2017 from 1249 patients. Naranjo score, modified Schumock and Thornton scale were used for causality and preventability assessments, respectively. Medication errors (MEs) were assessed by MEs tracking form. SPSS and Microsoft Excel were used for data analysis.

**Results:**

A total of 2686 antibiotics were prescribed to 1249 patients and 486 ADEs were found. The preventability assessment revealed that most of the ADEs (78.8%) were found among patients having long length of stay (LOS) in hospital and were preventable (59.3% of the ADEs were definitely preventable while 44.7% were probably preventable) and caused by MEs including wrong drug (40.1%) and monitoring errors (25%). The errors were caused due to non-adherence of policies (38.4%) and lack of information about antibiotics (32%). Most of the non-preventable ADEs or ADRs among patients having long and short LOS in hospital were “probable” (35.5%) and “possible” (35.8%), respectively. Logistic regression analysis revealed that ADEs were significantly less among females (OR = 0.047, 95% CI = 0.018─0.121, *p-value* = < 0.001), patients aged 18─52 years (OR = 0.041, 95% CI = 0.013─0.130, *p-value* = < 0.001), patients with ARTIs (OR = 0.004, 95% CI = 0.01–0.019, *p-value* = < 0.001), patients prescribed with 2 antibiotics per prescription (OR = 0.455, 95% CI = 0.319─0.650, *p-value* = < 0.001) and patients with long LOS (OR = 14.825, 95% CI = 11.198─19.627, *p-value* = < 0.001).

**Conclusion:**

Antibiotics associated definitely preventable ADEs were more commonly found in patients having long LOS in the inpatient departments because of MEs and lack of proper pharmacovigilance system. The ADRs showed a probable and possible causal association with both β-lactams and non β-lactams antibiotics.

## Background

According to the World Health Organization (WHO) adverse drug reactions (ADRs) are defined as, “any response to a drug which is noxious, unintended, and that occurs at doses normally used in man for the prophylaxis, diagnosis, or therapy of disease” [1]. On the basis of the National Coordinating Council for Medication Error Reporting and Prevention (NCC MERP) recommendations, adverse drug events (ADEs) can be termed as injuries which are either related to the medical interventions or the dose of the drugs [2]. As ADEs are not always associated with the use of drugs, so all ADRs can be attributed as ADEs but all ADEs can never be the result of ADRs. The risk of ADRs is associated with almost all the prescribed therapeutic agents. But these untoward effects may vary in terms of severity level i.e., from minor to severe or lethal [3].

The duration of a single episode of hospitalization i.e., length of stay (LOS) can be considered as one of the risk factors of ADEs. The stay of patient for each additional day in hospital increases the probability of developing ADEs because this provides more time for an ADE to occur [4]. According to a study if the LOS in hospital is prolonged then there will be an increment of 6% in the development of ADEs with the stay for each additional day [5]. Similarly, a Swedish study demonstrated ADRs as one of the most recurrent causes of mortality because one out of every seventh inpatient suffers from ADR during hospital stay [6]. A study demonstrated the prevalence rate of ADEs among hospitalized patients of England as 3.2%, Germany as 4.8% and the United States of America (USA) as 5.6% [7]. Furthermore, it is estimated that the incidence of life threatening ADRs during hospital stay ranges from 0.05 to 0.09% [8, 9]. Besides LOS, a meta-analysis revealed age, gender and drug exposure as the major contributing factors towards ADRs [8].

The prime role of pharmacovigilance system is to ensure patient safety due to its involvement in comprehension, recognition and prevention of ADEs [10]. The identification of ADEs still remains a major challenge for physicians. The causal association of ADRs with the drug is mandatory to evaluate in pharmacovigilance because it gives an insight about risk to benefit ratio of a particular drug on individual level [11]. Thus, poor monitoring and reporting system of ADEs has dramatically increase the patient’s LOS in hospital and economically burdened the healthcare system [12].

Antibiotics are among the most frequently prescribed therapeutic agents among hospitalized patients of all age groups [13]. It is estimated that more than half of the hospitalized patients are prescribed with antibiotics [14, 15]. It has been reported that the excessive use of antibiotics is associated with problems like antibiotic resistance [16]. Moreover, the higher rate of prescribing these agents has increased the chances of MEs up to several folds which in turn leads to the development of preventable ADEs [17]. In correspondence to this fact, a study conducted in Netherland report the incidence of preventable ADEs among 0.2% of the hospitalized patients [18]. A study conducted by Shehab, et al. documented that 19% patients visited emergency department due to antibiotics-associated ADRs [19]. Multiple reasons make inpatients more prone to ADRs which may include; 1) the trend of administering multiple antibiotics among inpatients. and 2) mostly, the inpatients comprises of pediatrics, geriatrics or patients having various co-morbidities and all these patients have high risk of developing ADRs [20, 21]. There is a dearth of proper pharmacovigilance surveillance system in Pakistan on regional, provincial and national level which leads to poor availability of data regarding antibiotic associated ADEs and its association with the LOS. Previously published studies do not give insight on this issue. The present study aims to assess the causality and preventability of adverse drug events of antibiotics (β-lactams and non β-lactams) among inpatients having different lengths of hospital stay.

## Methods

### Study design and settings

A prospective, cross-sectional, observational study was conducted in four public tertiary care hospitals (Mayo hospital, Jinnah hospital, General hospital, and Services hospital) of Lahore, Punjab province of Pakistan. According to latest Pakistani census, the total population living in Pakistan is 201,995,540 [22]. Lahore is the most populous city of Punjab province of Pakistan, with a total population of 11,126,285 [23]. The study settings lack pharmacovigilance centers and ADEs registers. The characteristics of the selected hospitals are summarized in Table 1.

**Table 1:** Characteristics of selected hospitals

Sr. no.	Characteristics	Mayo hospital	Jinnah hospital	General hospital	Services hospital
1	Number of beds	2400	1500	1300	1196
2	Inpatients visit last year	343, 114	217, 245	134, 491	125, 868
3	Prescribers/Medical officers	550	348	300	274
4	Nurses	500	313	271	249
5	Pharmacists/Dispensers	30	19	16	14
8	^a^ Other paramedical staff	671	445	382	304
10	Existence of pharmacovigilance center in hospital	No	No	No	No
11	Maintenance of ADR registers	No	No	No	No

^a^Other Paramedical staff includes; medical technicians, ward boys, and sweepers

### Study inclusion criteria

The study population included the patients of all age groups, admitted in general internal medicine ward and pediatric ward, prescribed with antibiotics on the basis of differential diagnosis for ≥24 h.

### Study exclusion criteria

All the patients with medical history of cardiac diseases, hepatic and renal insufficiencies, ear, nose and throat (ENT) disorders and unavailability of information regarding LOS in the hospital were excluded from this study.

### Data collection

A data collection form was developed which consisted of seven parts: 1) characteristics of the patients, 2) diagnosis, 3) recommended antibiotics, 4) medication errors, 5) causality assessment by Naranjo score, 6) preventability assessment and 7) the effect of ADRs on organ system (if any). The Anatomical Therapeutic Chemical (ATC) classification system [24] was used for the coding of antibiotics. SPSS version 21.0 was used for calculation of reliability coefficients. Internal consistency was measured by Cronbach’s alpha, while reproducibility was evaluated by using intra-class correlation for each item in the scales, with acceptable values ≥0.6. Calculation for Cronbach’s alpha was set at 0.76 for Schmuck and Thornton scale, 0.74 for ME tracking form, and 0.78 for Naranjo score. A pilot study was undertaken between November and December 2016 for pre-testing the study instrument. Data were collected between 1st January, 2017 and 30th June, 2017 according to the objectives of the study. The investigational team included a medical practitioner, pharmacist and a nurse. A total of 8 investigational teams were made. Two investigational teams were assigned to each hospital; one for internal medicine ward and other for pediatric ward.

The review of medical record was conducted on daily basis until the patient was discharged from the respective ward. This enables the investigators to scrutinize data from pertinent lab reports, physician’s progress notes, patient’s medication records (dose, dosage form, frequency and duration of prescribed antibiotics), physician’s order, multidisciplinary progress notes and discharge summaries. All the sign and symptoms that appeared after the use of antibiotics were also recorded. The team also participated in ward rounds and checked the presence of any alerts for MEs and ADEs. The expert opinions of physicians and clinical pharmacists were also taken in account before reaching the final decision about the occurrence of ADEs. The LOS in hospital was evaluated by measuring the difference between date of admission from the date of discharge [25]. Although it was difficult to evaluate whether the prolonged LOS in hospital was the contributing factor of ADEs or any underlying disease, so the assessment was made by taking into account the clinical judgments, nature and severity of underlying disease and social factors that may contribute in lengthening the patient’s stay time in hospital.

***Note****: In this study ADEs refers to injuries which are either caused by the drug (*i.e.*, ADRs or non-preventable ADEs) or by the use of the therapeutic agents (*i.e.*, medication errors or preventable ADEs) while ADRs refer to the definition given by Edwards and Aronson* i.e.*, unpleasant or harmful reactions that have causal relation with the medicinal product and predicts untoward outcomes from future administration and demands withdrawal from therapy, alteration of dosage regimen and specific treatments* [26]*. British National Formulary was used for confirming the ADRs* [27]*. MEs are those that occur during the processing of medication* i.e.*, prescribing, transcribing, dispensing, administering, adherence, or monitoring a drug* [28]*. MEs were identified through the standard guidelines of Current Medical Diagnosis AND Treatment (CMDT)* [29]*, National Institute of Health and Clinical Excellence (NICE) guidelines* [30]*, British National Formulary (BNF) for children* [31] *and Infectious Diseases Society of Pakistan (IDSP) guidelines for antibiotic use* [32]*.*

### Outcome variables

The outcome variables included causality assessment and preventability assessment. The cases in which ADEs appeared were further analyzed for assessing the preventability by Schumock and Thornton Scale. Medication errors were determined by using medication error tracking form among definitely preventable and probably preventable ADEs. Naranjo scale was used for determining the causal relationship between non-preventable ADEs and antibiotics.

#### Schumock and Thornton scale

The Schumock and Thornton criteria [33] was established for assessing the preventability of ADRs. The modified form of this criterion has been used in various studies [34, 35]. It has three sections namely definitely preventable, probably preventable and non-preventable. Section A comprises of five questions while section B has four questions. All the answers are categorized as “Yes” or “No”. ADRs were “definitely preventable” if answer was “yes” to one or more questions in section A. If answers were all negative then we proceeded to section B. ADRs were “probably preventable” if answer was “yes” to one or more questions in section B. If answers were all negative then we proceeded to section C. In Section C the ADRs were non-preventable.

#### Naranjo scale

The Naranjo Scale was developed by Naranjo and coworkers from the University of Toronto [36] for assessing the likelihood of whether an ADR is due to some particular drug or due to other factors. This validated tool has been used in multiple studies [37, 38]. This scale comprises of 10 questions that are answered “Yes”, “No”, or “Do not know”. Different point values (− 1, 0, + 1 or + 2) are assigned to each answer. Total scores range from − 4 to + 13; the reaction is considered definite if the score is 9 or higher, probable if 5 to 8, possible if 1 to 4, and doubtful if 0 or less.

#### Medication error tracking form

This tool was prepared for addressing MEs in hospitals for the California Health Care Foundation Data [39]. It consisted of three sections: 1) patient information, 2) medication order information and 3) medication error categorization. The third section comprised of “medication class”, “categories” and “possible causes” of MEs. It also classified MEs into five categories: A) prescribing, B) transcribing, C) dispensing, D) administering and E) monitoring.

### Statistical analysis

A convenient sampling technique was used to select the study participants. All the patients, admitted in internal medicine and pediatric departments during the 6 months of study period were considered as study population. Among them, patients met the inclusion criteria were taken as a sample size for this study. Statistical Package for Social Sciences (IBM Corp. Released 2012. IBM SPSS Statistics for Windows Version 21.0. Armonk, NY: IBM Corp.) and Microsoft Excel (MS Office 2010) were used for data analysis. Like previously published studies [40–42], descriptive statistics such as frequencies and percentages were used to present the data. And logistic regression analysis was performed to figure out the factors associated with ADEs. Results were expressed as Odds Ratio (OR) accompanied by 95% Confidence Intervals (95% CI) and a *p*-value < 0.05 was used for statistical significance of differences.

## Results

### Characteristics of the patients

According to hospitals records, 14,592 patients were admitted in internal medicine and pediatric departments during the 6 months of study period. A total of 1249 patients (age range 6 to 52 years) met the inclusion criteria of this study. Among them, 57.3% were male and 69.3% were aged > 18 years. 37% patients (*n* = 462) were prescribed antibiotics for urinary tract infections, 29% (*n* = 362) for acute respiratory tract infections, 23% (*n* = 287) for soft tissue infections and 11% (*n* = 137) for skin infections. Overall the LOS of 42.9% (*n* = 536) patients in the hospital was ≥5 days while 57.1% (*n* = 713) patients stayed for < 5 days in the healthcare settings (Table 2).

**Table 2:** Characteristics of patients (*N* = 1249)

Characteristics	n (%)^a^
Gender	
Male	716 (57.3)
Female	533 (42.7)
Age	
Adults (> 18 years)	865 (69.3)
Children (≤18 years)	384 (30.7)
Co-morbidities	
Diabetes	526 (42.1)
Asthma	424 (33.9)
Tuberculosis	137 (11.0)
Cystic fibrosis	162 (13.0)
Reasons of prescribing antibiotics	
Acute respiratory tract infections	362 (29.0)
Urinary tract infections	462 (37.0)
Soft tissue infections	287 (23.1)
Skin infections	138 (11.0)
Number of antibiotics prescribed per prescription	
1	229 (18.3)
2	603 (48.3)
3	417 (33.4)
LOS in the hospital	
Long (≥5 days)	536 (42.9)
Short (< 5 days)	713 (57.1)

^a^Percentages have been calculated with respect to the total sample size (*n* = 1249)

### Prescribing pattern of antibiotics

A total of 2686 antibiotics were prescribed among 1249 patients. Among β – Lactams, cephalosporins (10.9%, *n* = 292) while in non β – Lactams, fluoroquinolones (11.8%, *n* = 316) and macrolides (11.6%, *n* = 311) were the most frequently prescribed antibiotics (Table 3).

**Table 3:** Antibiotics prescribed among study population

Antibiotics Class	ATC code	Number of patients received antibiotics, *N* = 1249, n (%)	Number of prescribed antibiotics, *N* = 2686, n (%)
β – Lactams			
Penicillins	J01C	194 (15.5)	261 (9.7)
Carbapenem	J01DH	106 (8.5)	234 (8.7)
Cephalosporins	J01D	223 (17.9)	292 (10.9)
Non- β Lactams			
Flouroquinolones	J01 M	291 (23.3)	316 (11.8)
Aminoglycosides	J01G	192 (15.4)	226 (8.4)
Tetracyclines	J01AA	193 (15.5)	221 (8.2)
Lincosamide	J01FF	127 (10.2)	209 (7.8)
Macrolides	J01FA	252 (20.2)	311 (11.6)
Glycopeptide	J01XA	91 (7.3)	214 (7.9)
Oxazolidones	J01XX	102 (8.2)	186 (6.9)
Imidazole derivatives	G01AF	113 (9.5)	216 (8.0)

*ATC* Anatomical Therapeutic Chemical Classification System

### Organ system affected by ADEs

The proportion of ADEs was 486 (38.9%) among the total study participants. Overall, the most affected organ system by both β-lactams and non β-lactams antibiotics was GIT (long LOS = 35.8%, short LOS = 24.3%) as shown in Table 4.

**Table 4:** Effect of antibiotics on organ systems of study participants (*N* = 486)

Antibiotics	Total ADEs n (%)	LOS n (%)	Cardiac^a^ n (%)	GIT^b^ n (%)	Ototoxicity^c^ n (%)	Hematology^d^ n (%)	Hepatobiliary^e^ n (%)	Renal^f^ n (%)	Neurotoxicity^g^ n (%)	Others^h^ n (%)
β - Lactams										
Penicillins	62 (12.8)	Short LOS 0 (0.0)	0 (0.0)	0 (0.0)	0 (0.0)	0 (0.0)	0 (0.0)	0 (0.0)	0 (0.0)	0 (0.0)
		Long LOS 62 (100.0)	0 (0.0)	34 (54.8)	0 (0.0)	2 (3.2)	5 (8.1)	2 (3.2)	10 (16.1)	9 (14.5)
Carbapenem	34 (6.9)	Short LOS 9 (26.5)	0 (0.0)	2 (22.2)	0 (0.0)	0 (0.0)	7 (77.8)	0 (0.0)	0 (0.0)	0 (0.0)
		Long LOS 25 (73.5)	0 (0.0)	14 (56.0)	0 (0.0)	10 (40.0)	1 (4.0)	0 (0.0)	0 (0.0)	0 (0.0)
Cephalosporins	66 (13.6)	Short LOS 15 (22.7)	0 (0.0)	4 (26.7)	0 (0.0)	6 (40.0)	2 (13.3)	1 (6.7)	1 (6.7)	1 (6.7)
		Long LOS 51 (77.3)	0 (0.0)	10 (19.6)	0 (0.0)	18 (35.3)	17 (33.3)	5 (9.8)	1 (1.9)	0 (0.0)
*Total β – Lactams*	*162 (33.3)*	*Short LOS 24 (14.8)*	*0 (0.0)*	*6 (25.0)*	*0 (0.0)*	*6 (25.0)*	*9 (37.5)*	*1 (4.2)*	*1 (4.2)*	*1 (4.2)*
		*Long LOS 138 (85.2)*	*0 (0.0)*	*58 (42.0)*	*0 (0.0)*	*30 (21.7)*	*23 (16.7)*	*7 (5.1)*	*11 (7.9)*	*9 (6.5)*
Non- β Lactams										
Aminoglycosides	37 (7.6)	Short LOS 5 (13.5)	0 (0.0)	0 (0.0)	1 (20.0)	0 (0.0)	0 (0.0)	1 (20.0)	1 (20.0)	2 (40.0)
		Long LOS 32 (86.5)	0 (0.0)	5 (15.6)	10 (31.3)	2 (6.3)	0 (0.0)	10 (31.3)	2 (6.3)	3 (9.4)
Macrolides	61 (12.6)	Short LOS 5 (8.2)	0 (0.0)	1 (20.0)	1 (20.0)	1 (20.0)	1 (20.0)	0 (0.0)	0 (0.0)	1 (20.0)
		Long LOS 56 (91.8)	8 (14.3)	17 (30.4)	10 (17.9)	2 (3.4)	8 (14.3)	0 (0.0)	1 (1.8)	10 (17/9)
Fluoroquinolones	61(12.6)	Short LOS 17 (27.9)	0 (0.0)	0 (0.0)	6 (35.3)	2 (11.8)	6 (35.3)	1 (5.9)	0 (0.0)	2 (11.8)
		Long LOS 44 (72.1)	7 (15.9)	8 (18.2)	3 (6.8)	3 (6.8)	9 (20.5)	5 (11.4)	8 (18.2)	1 (2.3)
Tetracyclines	36 (7.4)	Short LOS 7 (19.4)	0 (0.0)	3 (42.9)	0 (0.0)	0 (0.0)	0 (0.0)	0 (0.0)	2 (28.6)	2 (28.6)
		Long LOS 29 (80.6)	0 (0.0)	16 (55.2)	0 (0.0)	0 (0.0)	0 (0.0)	0 (0.0)	4 (13.8)	9 (31.0)
Lincosamide	26 (5.4)	Short LOS 5 (19.2)	0 (0.0)	4 (80.0)	0 (0.0)	1 (20.0)	0 (0.0)	0 (0.0)	0 (0.0)	0 (0.0)
		Long LOS 21 (80.8)	0 (0.0)	15 (71.4)	0 (0.0)	3 (14.3)	0 (0.0)	0 (0.0)	0 (0.0)	3 (14.3)
Glycopeptide	37 (7.6)	Short LOS 18 (48.6)	0 (0.0)	4 (22.2)	0 (0.0)	0 (0.0)	0 (0.0)	8 (44.4)	0 (0.0)	6 (33.3)
		Long LOS 19 (51.4)	0 (0.0)	0 (0.0)	8 (42.1)	7 (36.8)	0 (0.0)	2 (10.5)	0 (0.0)	2 (10.5)
Oxazolidones	29 (5.9)	Short LOS 9 (31.0)	0 (0.0)	2 (22.2)	0 (0.0)	0 (0.0)	0 (0.0)	0 (0.0)	4 (44.4)	3 (33.3)
		Long LOS 20 (68.9)	0 (0.0)	7 (35.0)	0 (0.0)	1 (5.0)	0 (0.0)	0 (0.0)	5 (25.0)	7 (35.0)
Imidazole derivative	37 (7.6)	Short LOS 13 (35.1)	0 (0.0)	5 (38.5)	0 (0.0)	0 (0.0)	0 (0.0)	0 (0.0)	0 (0.0)	8 (61.5)
		Long LOS 24 (64.9)	0 (0.0)	11 (40.8)	0 (0.0)	0 (0.0)	0 (0.0)	0 (0.0)	13 (54.2)	0 (0.0)
*Total non β – Lactams*	*324 (66.7)*	*Short LOS 79 (24.4)*	*0 (0.0)*	*19 (24.1)*	*8 (10.1)*	*4 (5.1)*	*7 (8.9)*	*10 (12.7)*	*7 (8.9)*	*24 (30.4)*
		*Long LOS 245 (75.6)*	*15 (6.1)*	*79 (32.3)*	*31 (12.7)*	*18 (7.4)*	*17 (6.9)*	*17 (6.9)*	*33 (13.5)*	*35 (14.3)*
Total (*β – Lactams + Non β – Lactams)*	486 (38.9)	Short LOS 103 (21.2)	0 (0.0)	25 (24.3)	8 (7.8)	10 (9.7)	16 (15.5)	11 (10.7)	8 (7.8)	25 (24.3)
		Long LOS 383 (78.8)	15 (3.9)	137 (35.8)	31 (8.1)	48 (12.5)	40 (10.4)	24 (6.3)	44 (11.5)	44 (11.5)

^a^QTc > 440 millisecond (ms) in males or > 460 ms in females in the absence of preexisting arrhythmias, based on ≥2 electrocardiograms

^b^Abdominal discomfort, nausea and vomiting associated with antibiotic administration, in the absence of an alternate explanation

^c^The ability of speech discrimination was diminished upon administration of antibiotics

^d^Developed in the absence of myelosuppressive drugs and characterized as thrombocytopenia (decrease in platelet count < 150 × 103/μL), anemia (decrease in hemoglobin level < 10 g/dL) and leukopenia (decrease in white blood cells level < 4500 cells/ μL)

^e^Characterized as increase in total bilirubin (> 3 mg/dL) or alanine transaminase (> 3 times patient’s baseline) or aspartate transaminase (> 3 times patient’s baseline) when there was no preexisting hepatobiliary disease

^f^Characterized as high level of serum creatinine i.e. > 1.5 time baseline when there was no preexisting acute kidney injury (e.g. sepsis) or exposure to nephrotoxic drug or intravenous contrast

^g^Demonstrated as antibiotic associated toxicity, peripheral neuropathy, seizures (when there was no preexisting neurologic condition) or altered mental condition

^h^Other ADRs among children may include penicillins-associated hypersensitivity; macrolides-associated rashes and Stevens-Johnson syndrome; flouroquinolones-associated arthralgia and tendon disorders; tetracyclines-associated tooth discoloration and enamel defects; Lincosamide-associated metallic taste; Glycopeptide-associated flushing and maculopapular rash; Oxazolidones-associated red man syndrome, pruritus and oral candidiasis; imidazole-associated taste disturbance. Other ADRs among adults may include penicillins-associated hypersensitivity; aminoglycosides-associated stomatitis; macrolides-associated pancreatitis; cephalosporins-associated Stevens-Johnson syndrome, pruritus and urticaria; Fluoroquinolones-associated hypotension; Tetracyclines-associated rash, dermatitis and angioedema; Glycopeptide-associated red man syndrome and phlebitis; Oxazolidones-associated taste disturbance and polyuria; imidazole-associated taste disturbance and neuropathy

### Preventability assessment

More than half (*n* = 383, 78.8%) of the ADEs were found among patients having long LOS in hospital. Among them, most of the ADEs were preventable i.e., the proportion of definitely preventable ADEs was 171 (44.7%); whereas, the proportion of probably preventable ADEs was 56 (14.6%) according to modified Schumock and Thornton criteria (Table 5).

**Table 5:** Preventability assessment (*N* = 486)

Schumock and Thornton criteria	Long LOS, *N* = 383, n (%)	Short LOS, *N* = 103, n (%)	Total, *N* = 486, n (%)
Section A: Definitely preventable ADEs			
Was there a history of allergy or previous reaction to the drug?	4 (1.0)	5 (4.9)	9 (1.9)
Was the drug involved inappropriate for the patient’s clinical condition?	100 (26.1)	14 (13.6)	114 (23.5)
Was the dose, route, or frequency of administration inappropriate for patient’s age, weight or disease state?	53 (13.8)	11 (10.7)	64 (13.2)
Was toxic serum drug concentration or lab monitoring test documented?	7 (1.8)	9 (8.7)	16 (3.3)
Was there a known treatment for ADEs?	7 (1.8)	2 (1.9)	9 (1.9)
*Total*	*171 (44.7)*	*41 (39.8)*	*212 (43.6)*
Section B: Probably preventable ADEs			
Was therapeutic drug monitoring or other necessary lab test not performed?	31 (8.1)	7 (6.8)	38 (7.8)
Was the drug interaction involved in ADEs?	4 (1.0)	2 (1.9)	6 (1.2)
Was poor compliance involved in ADE?	13 (3.4)	4 (3.9)	17 (3.5)
Were preventative measures not prescribed or administered to the patient?	8 (2.1)	3 (2.9)	11 (2.3)
*Total*	*56 (14.6)*	*16 (15.5)*	*72 (14.8)*
*Total (preventable ADEs)*	*227 (59.3)*	*57 (55.3)*	*284 (58.4)*
Section C: Non-preventable ADEs or ADRs			
If all the above criteria not fulfilled.	156 (40.7)	46 (44.7)	202 (41.6)

*LOS* Length of stay *ADEs* Adverse drug events *ADRs* Adverse drug reactions

Overall most of the definitely preventable (63.7%, *n* = 109), probably preventable (69.6%, *n* = 39) and non-preventable ADEs (62.2%, *n* = 92) were most commonly caused by non β-Lactams as compared to β-Lactams class of antibiotics especially among patients having long LOS in hospital (Table 6).

**Table 6:** Adverse drug events with respect to class of prescribed antibiotics (*N* = 486)

Antibiotics	ATC code	Definitely preventable ADEs			Probably preventable ADEs			Non-preventable ADEs		
		Long LOS, *N* = 171, n (%)	Short LOS, *N* = 41, n (%)	Total, *N* = 212, n (%)	Long LOS, *N* = 56, n (%)	Short LOS, *N* = 16, n (%)	Total, *N* = 72, n (%)	Long LOS, *N* = 156, n (%)	Short LOS, *N* = 46, n (%)	Total, *N* = 202, n (%)
β - Lactams										
Penicillins	J01C	25 (14.6)	0 (0.0)	25 (11.8)	9 (16.1)	0 (0.0)	9 (12.5)	28 (17.9)	0 (0.0)	28 (13.9)
Carbapenem	J01DH	11 (6.4)	7 (17.1)	18 (8.5)	2 (3.6)	0 (0.0)	2 (2.8)	12 (7.7)	2 (4.4)	14 (6.9)
Cephalosporins	J01D	26 (15.2)	5 (12.2)	31 (14.6)	6 (10.7)	0 (0.0)	6 (8.3)	19 (12.2)	10 (21.7)	29 (14.4)
*Total β – Lactams*		*62 (36.2)*	*12 (29.3)*	*74 (34.9)*	*17 (30.4)*	*0 (0.0)*	*17 (23.6)*	*59 (37.8)*	*12 (26.1)*	*71 (35.2)*
Non- β Lactams										
Flouroquinolones	J01 M	18 (10.5)	3 (7.3)	21 (9.9)	7 (12.5)	6 (37.5)	13 (18.1)	19 (12.2)	8 (17.4)	27 (13.4)
Aminoglycosides	J01G	13 (7.6)	3 (7.3)	16 (7.6)	6 (10.7)	0 (0.0)	6 (8.3)	13 (8.3)	2 (4.4)	15 (7.4)
Tetracyclines	J01AA	12 (7.0)	1 (2.4)	13 (6.1)	5 (8.9)	3 (18.8)	8 (11.1)	12 (7.7)	3 (6.5)	15 (7.4)
Macrolides	J01FA	27 (15.8)	4 (9.8)	31 (14.6)	4 (7.1)	0 (0.0)	4 (5.6)	25 (16.0)	1 (2.2)	26 (12.9)
Lincosamide	J01FF	4 (2.3)	1 (2.4)	5 (2.4)	10 (17.8)	0 (0.0)	10 (13.9)	7 (4.5)	4 (8.7)	11 (5.5)
Glycopeptide	J01XA	9 (5.3)	8 (19.5)	17 (8.0)	2 (3.6)	2 (12.5)	4 (5.6)	8 (5.1)	8 (17.4)	16 (7.9)
Oxazolidones	J01XX	13 (7.6)	3 (7.3)	16 (7.6)	1 (1.8)	3 (18.8)	4 (5.6)	6 (3.9)	3 (6.5)	9 (4.5)
Imidazole derivatives	G01AF	13 (7.6)	6 (14.6)	19 (8.9)	4 (7.1)	2 (12.5)	6 (8.3)	7 (4.5)	5 (10.9)	12 (5.9)
*Total Non β – Lactams*		*109 (63.7)*	*29 (70.7)*	*138(65.1)*	*39 (69.6)*	*16(100.0)*	*55 (76.4)*	*97 (62.2)*	*34 (73.9)*	*131(64.9)*

*ATC* Anatomical Therapeutic Chemical Classification System, *ADEs* Adverse drug events, *LOS* Length of stay

### Medication errors

Among 284 cases of preventable ADEs, the wrong drug errors (*n* = 114, 40.1%) and monitoring errors (*n* = 71, 25%) were more commonly found among study population. The antibiotics administered through oral route had greater ADEs (proportion of ADEs = 4 out of 5) as compared to the antibiotics administered through parental route (proportion of ADEs = 1 out of 5). Physician ordering (22.2%, *n* = 63) and patient monitoring (21.1%, *n* = 60) were the most common stages of medication errors. These errors were caused due to non-adherence of policies and procedures (38.4%, *n* = 109) and lack of information about antibiotics (32%, *n* = 91) (Table 7).

**Table 7:** Antibiotic associated errors in study population (*N* = 284)

Variables	Long length of stay, *N* = 227, n (%)	Short length of stay, *N* = 57, n (%)	Total, *N* = 284, n (%)
Type of medication errors			
Wrong drug	100 (44.1)	14 (24.6)	114 (40.1)
Wrong dose	35 (15.4)	6 (10.5)	41 (14.4)
Wrong route	2 (0.9)	3 (5.3)	5 (1.8)
Wrong time	13 (5.7)	2 (3.5)	15 (5.3)
Deteriorated drug	3 (1.3)	0 (0.0)	3 (1.1)
Omission	12 (5.3)	3 (5.3)	15 (5.3)
Wrong dosage form	3 (1.3)	0 (0.0)	3 (1.1)
Non-adherence	13 (5.7)	4 (7.0)	17 (5.9)
Monitoring error	46 (20.3)	25 (43.9)	71 (25.0)
Stages of errors			
Physician ordering	59 (25.9)	4 (7.0)	63 (22.2)
Transcribing	41 (18.1)	7 (12.3)	48 (16.9)
Dispensing pharmacist	36 (15.9)	14 (24.6)	50 (17.6)
Nurse administering	37 (16.3)	9 (15.8)	46 (16.2)
Patient monitoring	37 (16.3)	23 (40.4)	60 (21.1)
Others^a^	17 (7.5)	0 (0.0)	17 (5.9)
Causes of errors			
Lack of knowledge about the patients^b^	46 (20.3)	2 (3.5)	48 (16.9)
Lack of information about antibiotics^c^	77 (33.9)	14 (24.6)	91 (32.0)
Non-adherence to policies and procedures^d^	73 (32.2)	36 (63.2)	109 (38.4)
Miscellaneous^e^	31 (13.7)	5 (8.8)	36 (12.7)

^a^Medication errors due to patient non-adherence

^b^Information about allergy, lab tests results, concomitant medications and conditions either not available or noted

^c^Indication for antibiotic use, compatibility, available dosage form, dosing guidelines and route of administration

^d^Use of abbreviation in medication ordering, incomplete medication order processed, deviation from treatment protocols, delay in dispensing, use of non-standard dosing schedule, and drug preparation errors

^e^Illegible handwriting of physicians, memory lapse, and unavailability of drugs

### Causality assessment

156 (77.2%) ADEs were detected among patients having long LOS (> 5 days) and 46 (22.3%) among patients having short LOS (≤ 5 days). Overall, most of the ADRs were “probable” (long LOS = 35.3%, short LOS = 34.8%) and “possible” (long LOS = 33.9%, short LOS = 30.4%) and occurred more frequently due to non β lactams as compared to β lactams antibiotics (Table 8).

**Table 8:** Causality assessment with respect to antibiotics class (*N* = 202)

Antibiotics Class	ATC code	Long length of stay					Short length of stay				
		Naranjo score				Total ADRs	Naranjo score				Total ADRs
		Definite^a^ n (%)	Probable^b^ n (%)	Possible^c^ n (%)	Doubtful^d^ n (%)		Definite^a^ n (%)	Probable^b^ n (%)	Possible^c^ n (%)	Doubtful^d^ n (%)	
β – Lactams											
Penicillins	J01C	1 (3.6)	15 (53.6)	4 (14.3)	8 (28.6)	28	0 (0.0)	0 (0.0)	0 (0.0)	0 (0.0)	0
Carbapenem	J01DH	0 (0.0)	5 (41.7)	3 (25.0)	4 (33.3)	12	1 (50.0)	1 (50.0)	0 (0.0)	0 (0.0)	2
Cephalosporins	J01D	0 (0.0)	7 (36.8)	9 (47.4)	3 (15.8)	19	3 (30.0)	1 (10.0)	1 (10.0)	5 (50.0)	10
*Total β - Lactams*		*1 (1.7)*	*27 (45.8)*	*16 (27.1)*	*15 (25.4)*	*59*	*4 (33.3)*	*2 (16.7)*	*1 (8.3)*	*5 (41.7)*	*12*
Non- β Lactams											
Flouroquinolones	J01 M	4 (21.1)	6 (31.6)	7 (36.8)	2 (10.5)	19	0 (0.0)	5 (62.5)	2 (25.0)	1 (12.5)	8
Aminoglycosides	J01G	2 (15.4)	4 (30.8)	5 (38.5)	2 (15.4)	13	0 (0.0)	1 (50.0)	0 (0.0)	1 (50.0)	2
Macrolides	J01FA	2 (7.1)	5 (20.0)	12 (48.0)	6 (24.0)	25	0 (0.0)	0 (0.0)	1 (100.0)	0 (0.0)	1
Tetracyclines	J01AA	0 (0.0)	3 (25.0)	4 (33.3)	5 (41.7)	12	2 (66.7)	0 (0.0)	1 (33.3)	0 (0.0)	3
Lincosamide	J01FF	0 (0.0)	2 (28.6)	2 (28.6)	3 (42.9)	7	1 (25.0)	3 (75.0)	0 (0.0)	0 (0.0)	4
Glycopeptide	J01XA	2 (25.0)	3 (37.5)	1 (12.5)	2 (25.0)	8	0 (0.0)	2 (25.0)	5 (62.5)	1 (12.5)	8
Oxazolidones	J01XX	0 (0.0)	2 (33.3)	4 (66.7)	0 (0.0)	6	0 (0.0)	1 (33.3)	1 (33.3)	1 (33.3)	3
Imidazole derivatives	G01AF	1 (14.3)	3 (42.9)	2 (28.6)	1 (14.3)	7	0 (0.0)	2 (40.0)	3 (60.0)	0 (0.0)	5
*Total Non β - Lactams*		*11 (11.3)*	*28 (28.9)*	*37 (38.1)*	*21 (21.7)*	*97*	*3 (8.8)*	*14 (41.2)*	*13 (38.2)*	*4 (11.8)*	*34*
Total (*β – Lactams + Non β – Lactams)*		12 (7.7)	55 (35.3)	53 (33.9)	36 (23.1)	156	7 (15.2)	16 (34.8)	14 (30.4)	9 (19.6)	46

^a^Definite (≥ 9 score) ADRs are (1) followed a chronological sequence after the administration of drug or in which the drug had achieved a toxic concentration in the tissues or physiological fluid, and (3) could show improvement when the drug was withdrawal but reappeared on exposure

^b^Probable (5–8 score) ADRs are (1) followed a chronological sequence after the administration of drug, (2) were in accordance to a recognized pattern of reactions, (3) were not confirmed by the exposure to the suspected drug but by the withdrawal of that drug, and (4) could not be described by features of the patient’s disease

^c^Possible (1–4) ADRs are (1) could be described by features of the patient’s disease, (2) followed a chronological sequence after the administration of drug, and (3) were in accordance to a recognized pattern of reactions

^d^Doubtful (≤0) are factors other than a drug are associated with the reactions

### Determinants associated with ADEs among study respondents

Logistic regression analysis was used to examine the association between ADEs and the independent variables. Results of this analysis revealed that females had 95.3% less ADEs (OR = 0.047, 95% CI = 0.018─0.121, *p-value* = < 0.001) as compared to males. Among the age groups, patients aged > 18 years (OR = 0.041, 95% CI = 0.013─0.130, *p-value* = < 0.001) were likely to have less ADEs as compared to patients aged ≤18 years. While examining the association between co-morbidities and ADEs, asthmatic patients (OR = 0.808, 95% CI = 0.598─1.093, *p-value* = 0.167), tuberculosis patients (OR = 0.304, 95% CI = 0.186─0.497, *p-value* = < 0.001) and cystic fibrosis patients (OR = 0.527, 95% CI = 0.334─0.829, *p-value* = 0.006) were likely to have less ADEs as compared to diabetic patients. According to diagnosis, patients with acute respiratory tract infections had 99.6% less ADEs (OR = 0.004, 95% CI = 0.01–0.019, *p-value* = < 0.001) and patients with soft tissue infections had 95.1% less ADEs (OR = 0.49, 95% CI = 0.018–0.133, *p-value* = < 0.001) as compared to the patients having urinary tract infections. Among the number of antibiotics prescribed per prescription, 2 antibiotics prescribed per prescription had 54.5% less ADEs (OR = 0.455, 95% CI = 0.319─0.650, *p-value* = < 0.001) while 3 antibiotics prescribed per prescription had 1.529 times more ADEs (OR = 1.529, 95% CI = 1.063─2.198, *p-value* = 0.022) as compared to those which had 1 antibiotic prescribed per prescription. According to LOS in hospital, patients with long LOS had 14.825 times more ADEs (OR = 14.825, 95% CI = 11.198─19.627, *p-value* = < 0.001) as compared to patients who had short LOS (Table 9).

**Table 9:** Logistic regression analysis of factors associated with Adverse drug events (*N* = 1249)

Characteristics	ADEs		*OR*	*95% CI*	*p-value*
	Yes n (%)	No n (%)			
Gender					
Male	293 (23.5)	423 (33.9)	1.0	–	–
Female	193 (15.5)	340 (27.2)	0.047	0.018─0.121	**< 0.001**
Age					
Children (≤18 years)	184 (14.7)	200 (16.0)	1.0	–	–
Adults (> 18 years)	302 (24.2)	563 (45.1)	0.041	0.013─0.130	**< 0.001**
Co-morbidities					
Diabetes	210 (16.8)	316 (25.3)	1.0	–	–
Asthma	169 (13.5)	255 (20.4)	0.808	0.598─1.093	0.167
Tuberculosis	37 (3.0)	100 (8.0)	0.304	0.186─0.497	**< 0.001**
Cystic fibrosis	70 (5.6)	92 (7.4)	0.527	0.334─0.829	**0.006**
Reasons of prescribing antibiotics					
Urinary tract infections	198 (15.9)	264 (21.1)	1.0	–	–
Acute respiratory tract infections	157 (12.6)	205 (16.4)	0.004	0.001─0.019	**< 0.001**
Soft tissue infections	131 (10.5)	156 (12.5)	0.049	0.018─0.133	**< 0.001**
Skin infections	0 (0.0)	138 (11.0)	0.000	0.000─0.000	0.994
Number of antibiotics prescribed per prescription					
1	101 (8.1)	128 (10.2)	1.0	–	–
2	153 (12.2)	450 (36.0)	0.455	0.319─0.650	**< 0.001**
3	232 (18.6)	185 (14.8)	1.529	1.063─2.198	**0.022**
LOS					
Short (< 5 days)	103 (30.7)	610 (48.8)	1.0	–	–
Long (≥5 days)	383 (8.2)	153 (12.2)	14.825	11.198─19.627	**< 0.001**

*ADEs* Adverse drug events, *OR* Odd Ratio, *CI* Confidence Interval, *LOS* Length of stay. The variables with *p*-value <0.05 are significantly associated with adverse drug events

## Discussion

The current study set out to determine the causality and preventability of ADEs associated with the use of antibiotics among inpatients having different LOS in hospital. It was revealed that overall 38.9% of the patients were detected with ADEs upon administering β-lactams and non β-lactams antibiotics. ADRs were less commonly observed as compared to preventable ADEs. MEs especially wrong drug selection might be the possible reason for preventable ADEs. The disobedience of international guidelines and non-availability of national formularies are the biggest hurdles in provision of optimal patient care and thus raise the risk of inappropriate prescribing and MEs [43–48]. This finding is in line with a previously published study where inappropriate prescribing trend of antibiotics had been the cause of majority of the non-preventable ADEs [49]. A study conducted in an Indian healthcare setting also predicted that less than half of the total ADEs were non-preventable and caused by β-lactams. Most of these non-preventable ADEs had a probable or possible causal relationship with the antibiotics [50]. In Pakistan, the high rate of preventable ADEs is the result of several factors which mainly include, (a) non-availability of clinical pharmacist during ward rounds and prescription evaluation, (b) improper monitoring and reporting of ADRs, ADEs and MEs due to unestablished pharmacovigilance centers, (c) high patient load in public hospitals, and (d) low budget allocation for healthcare systems by the government [51–56].

Findings also suggested that ADEs associated with the use of β-lactams and non β-lactams antibiotics had mainly affected the GIT, hematologic system and skin. These results are in line with the previously published studies that predict the GIT as the most affected organ system by the antibiotic associated ADEs [57, 58]. The prime reason of it might be the suppression of normal flora of gut upon oral administration of antibiotics that may lead to the pathogenic and non-pathogenic colonization in GIT [59]. Thus, it is the need of the hour to establish a proper pharmacovigilance surveillance system under Drug Regulatory Authority of Pakistan (DRAP) for the proper monitoring and reporting of ADEs in all the primary, secondary and tertiary care settings. This initiative of provincial and federal government will be fruitful in making statistical analysis of ADEs at national level.

Like the previously published studies [58, 60], most of the non-preventable ADEs or ADRs were “probable” and observed in patients having long LOS in hospital. As the under lying diseases may lead to poly pharmacy, so an ADR cannot be designated to have a definite causal association with the single therapeutic agent [61]. The causality assessment of antibiotics with ADRs is helpful in providing optimal care, establishing safety measures and preventing the risk of reoccurrence and iatrogenic complications [62].

The statistically significant association was established between ADEs and several risk factors by using logistic regression analysis. These factors mainly include age, gender, co-morbidities, number of drugs which were being exposed to the patient and LOS in hospital. It was found that factors like adult age group, female patients, under lying diseases (tuberculosis and acute respiratory infections), prescribing 2 antibiotics per prescription and short LOS (> 5 days) were significantly less associated with the development of ADEs. Similar association of age and prescribing antibiotics with ADEs was also significantly found in a previously published study [63]. The physiological and pharmacological differences may cause drugs to respond differently among different age groups [64]. Another study revealed significant association of ADEs with the number of drugs exposed but insignificant association was found with the age and gender [65]. As the risk of drug interactions is directly proportional to the number of drugs prescribed per prescription, so it may lead to the development of ADEs [66]. The significant correlation of male gender with the development of ADEs found in the present study is also in line with the previously published literature [50, 67]. This is in contrast with other studies which showed significant correlation of female gender with the development of ADEs [68, 69]; however, some studies declared no significant association of ADEs with gender [65, 70]. The prime reason of this divergence is that besides biologic differences several social, behavioral, cultural and physiological dissimilarities may have an impact on factors like gender [71]. It was also found that co-morbidities like diabetes mellitus (DM) had a significant association with ADEs because this metabolic disease may negatively affect the renal function and cause the undesired metabolism of drugs which makes the patients more prone towards the development of ADEs [72]. Long LOS in hospital is also found to be significantly associated with the ADEs which is consistent with the results of previously study wherein most of the preventable ADEs caused an increase in LOS in healthcare settings [73–77]. This is because of the fact that more number of prescribed drugs may increases the risk of drug interactions and MEs and thus leading to increased LOS in the healthcare settings.

This study has some limitations. First, the effects of prescribing multiple drugs at the same time or switching between drugs (bacterial resistance, or changing the medication after a full course treatment) have not been determined. Second, since the data was collected for short period of time and no follow up could be performed after the discharge of patients, so the long term effects of ADEs on organ systems (e.g., liver and kidney) and its risk factors could not be determined. However, future longitudinal studies could address these aspects. Third, the outcomes of treatment interventions like rechallenge and dechallenge were not measured in this study, therefore very few had shown a definite causal association of ADEs with the antibiotics. Last, the Hawthorne effect could have affected the result because physicians, nurses and other paramedical staff were well aware of the study.

## Conclusion

The current study concluded that non β-lactams were among the most frequently prescribed antibiotics and most of the ADEs caused an increase in LOS in hospital. As per preventability assessment, most of the ADEs were preventable because these were caused due to MEs during the stages of medication processing like physician ordering and patient monitoring. Most of the non-preventable ADEs were having probable causal relationship with the antibiotics and found in patients having prolonged LOS. Gastrointestinal system, hematologic system and skin rashes were commonly found in patients prescribed with both β-lactams and non β-lactams. Moreover, the logistic regression showed significant association between ADEs and its risk factors like age, gender, co-morbidities, number of prescribed antibiotics and LOS in hospital. The present findings are beneficial as they give an insight about the current pharmacovigilance system and open the doorsteps for stakeholders in making strategies to overcome these issues.

## Abbreviations

ADEs: Adverse drug events
ADRs: Adverse drug reactions
DRAP: Drug Regulatory Authority of Pakistan
LOS: Length of stay
MEs: Medication errors
WHO: World Health Organization
